# Hydroacoustics as a tool to examine the effects of Marine Protected Areas and habitat type on marine fish communities

**DOI:** 10.1038/s41598-017-18353-3

**Published:** 2018-01-15

**Authors:** J. P. Egerton, A. F. Johnson, J. Turner, L. LeVay, I. Mascareñas-Osorio, O. Aburto-Oropeza

**Affiliations:** 1 School of Ocean Sciences, Bangor University, Menai Bridge, Wales UK; 2 Marine Biology Research Division, Scripps Institution of Oceanography, La Jolla, CA United States of America; 3 Centro para la Biodiversidad y Conservación, La Paz, Mexico

## Abstract

Hydroacoustic technologies are widely used in fisheries research but few studies have used them to examine the effects of Marine Protected Areas (MPAs). We evaluate the efficacy of hydroacoustics to examine the effects of closure to fishing and habitat type on fish populations in the Cabo Pulmo National Park (CPNP), Mexico, and compare these methods to Underwater Visual Censuses (UVC). Fish density, biomass and size were all significantly higher inside the CPNP (299%, 144% and 52% respectively) than outside in non-MPA control areas. These values were much higher when only accounting for the reefs within the CPNP (4715%, 6970% and 97% respectively) highlighting the importance of both habitat complexity and protection from fishing for fish populations. Acoustic estimates of fish biomass over reef-specific sites did not differ significantly from those estimated using UVC data, although acoustic densities were less due to higher numbers of small fish recorded by UVC. There is thus considerable merit in nesting UVC surveys, also providing species information, within hydroacoustic surveys. This study is a valuable starting point in demonstrating the utility of hydroacoustics to assess the effects of coastal MPAs on fish populations, something that has been underutilised in MPA design, formation and management.

## Introduction

Marine Protected Areas (MPAs) have been suggested as one of a suite of spatial management tools attempting to reduce the pressures posed by anthropogenic threats on marine life and habitats^1,2^. Whilst MPAs may be designated for a variety of reasons^3,4^ one common objective is the protection of exploited fish populations^5^. Consequently, there have been many studies evaluating the efficacy of MPAs in protecting fish populations and recent meta-analyses report net positive increases of fish density, diversity, body-size and biomass^6–11^ within MPAs. Developing suitable fish population monitoring programmes to evaluate MPA success is, however, often a difficult task^12^.

Most studies on the response of fish populations to different levels of fishing intensity or management regime overlook pelagic species and tend to focus on less mobile demersal species for which there are stronger links with bottom habitat types^7,13,14^. Such studies tend to use survey techniques such as trapping, fishing, camera recordings and Underwater Visual Census (UVC). Fish survey methods that provide more detail on the mid-water component within MPAs may also reveal how pelagic species and the benthic-pelagic coupling respond to protection, an area of research that comparatively, is lacking^13,15,16^. In this respect, active hydroacoustics have the advantage that they can sample almost the entire water column^17^, whereas Underwater Visual Census (UVC) is focussed predominantly on demersal species (i.e. from the seabed to a given height above it). Hydroacoustics can also cover a much greater area per unit of time, allowing large spatial scales to be studied which may be necessary to sample highly mobile species. The relatively fast data acquisition of hydroacoustic methods also adds to the time-saving (and therefore often cost-saving) benefits when compared to alternative fish survey methods^18^, and data are immediately digitally recorded following acquisition^19^. Hydroacoustic fish survey methods also have the advantage that they are non-destructive in nature and are not hampered by issues such as water clarity, strong currents or diver depth limits. Hydroacoustic methods do, however, require groundtruthing to gain species-specific information and for the most accurate calculations of fish lengths and weight^20^.

Although hydroacoustic surveys offer many advantages over other fish survey methods, we are unaware of any published research using hydroacoustics to evaluate the effects of both protection regime and habitat type on marine fish populations. Egerton *et al*.^21^ used hydroacoustics to locate and quantify Nassau grouper spawning aggregations within MPAs, but did not examine the effects of protection *per se*. Polunin *et al*.^22^ found that acoustic surveys can provide a cost-efficient method of assessing fish biomass within an MPA in comparison to other methods (baited traps, baited video and trammel nets), but did not aim to use these different methods to compare fish communities inside vs outside their protected study area. Similarly, Rudershausen *et al*.^23^ used acoustics and fish traps in an MPA off the South-eastern US coast, with the aim of comparing the two methods rather than examining the effect of protection regime or habitat type on the local fish populations. Habitat type is well understood to have a significant influence on fish community composition and distribution^24,25^. In order to comprehensively evaluate the effects of marine protection on fish populations, seabed habitat type therefore needs to be taken into account^8,26^. Most studies demonstrate increases in fish abundance^27^ and biomass^28,29^ with increasing habitat complexity. Many studies evaluating the effects of MPAs, however, often fail to consider such habitat effects, which, in some cases may mask the effects of protection considerably^13,25^. Although abiotic and biotic habitat often has an influence on fish community composition^30^, our focus in this study was the abiotic habitat and throughout this study we use the word ‘habitat’ synonymously with ‘substrate’^31^.

**Figure 1:**
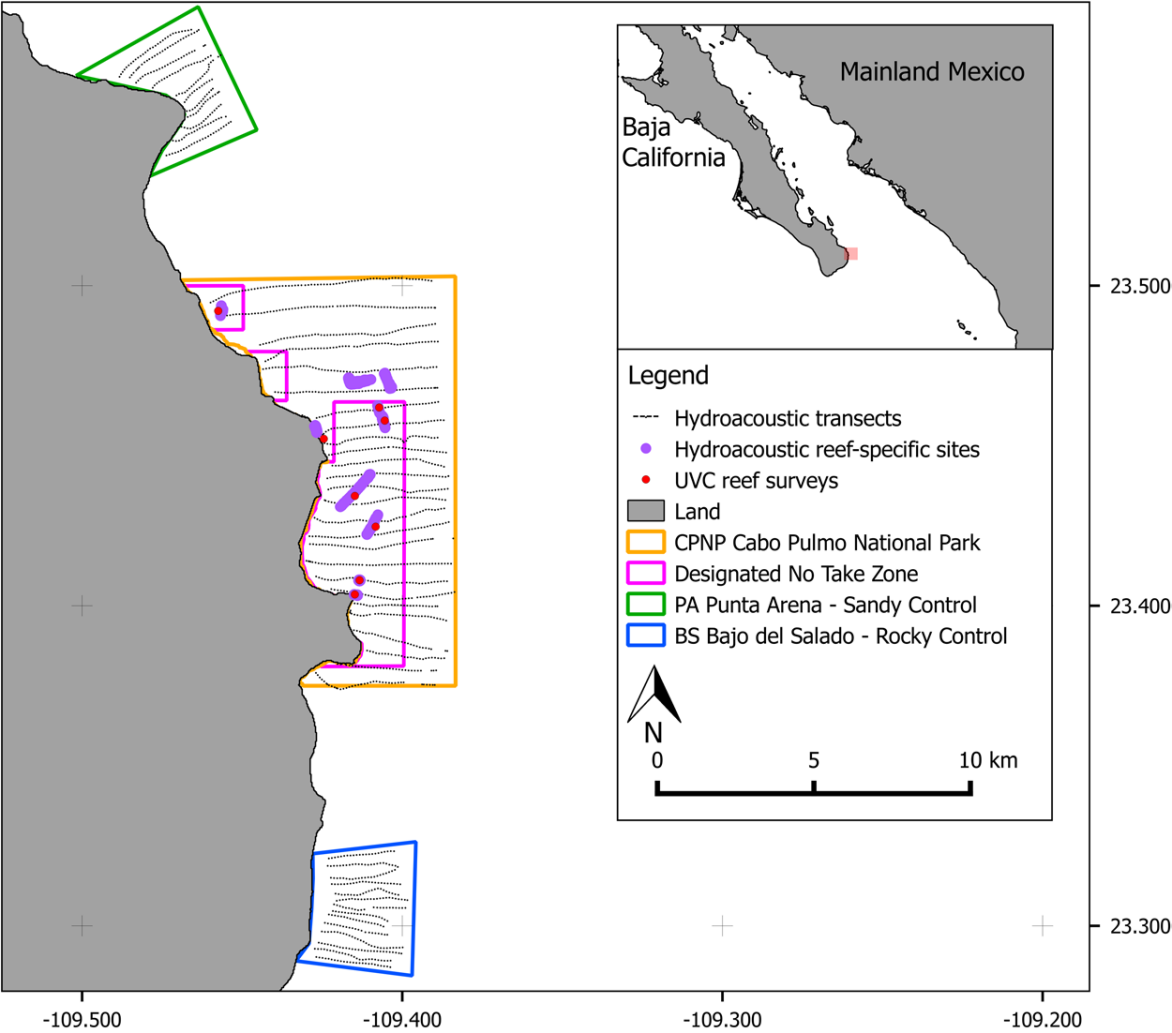
Location of the survey sites and hydroacoustic transects at Cabo Pulmo, Baja California Sur, Mexico. Also shown is the core No Take Zone within the park and the locations of the Underwater Visual Census (UVC) sites. Coordinates are in WGS84. Map generated in Quantum GIS ver 2.18 (www.qgis.org).

The Cabo Pulmo National Park (CPNP), Baja California Sur, Mexico was established in 1995, with considerable involvement from the local community^32^, and covers an area of 7,111 hectares^33^ (Fig. 1). A major factor governing the success of an MPA is how well it has been enforced^34^. Although only 35% of the CPNP is designated as a ‘no take’ area, the local community follow and enforce a policy of no-fishing throughout the entire reserve^35^. Fourteen years following the creation of the park, a long-term ecological monitoring program employing UVC surveys reported a 463% increase in fish biomass^36^. This is in keeping with a mean biomass increase of 446% reported in a meta-analysis of 55 MPAs globally^8^. The CPNP is composed of a mixture of habitats with basaltic rocky reef dikes forming long, parallel ridges that run adjacent to shore in the northern section of the park, while disappearing under the shoreline in the south-central section^37^. Isolated coral heads grow on top of these ridges, and the highest amount of coral cover is around 15–20% over central sections^38^. Between the rocky reefs the seafloor habitats consist primarily of sand interspersed with sparse boulder fields.

Past fish population surveys of the CPNP have employed teams of SCUBA divers surveying linear transects along the rocky reefs of the park counting fish and invertebrates, estimating mean sizes of individual and schooling fish. In this study we use a split beam echosounder to conduct hydroacoustic surveys to evaluate the effects of protection from fishing and habitat type by examining the total fish density, total fish biomass and mean fish size within the CPNP in comparison to sites outside the park. Further, the hydroacoustic ‘reef-specific’ transects that specifically targeted the reefs within the park are compared with the belt transect UVC estimations carried out over corresponding reef sites in the same year. Finally, we comment on the efficacy of using hydroacoustic surveys to measure the effects of protection and habitat type on fish populations.

## Results

### Acoustic fish density

**Figure 2:**
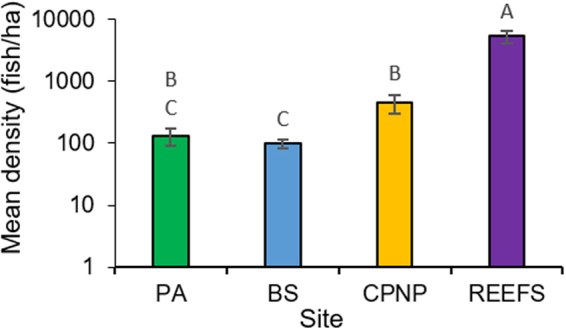
Acoustic Mean density of fish/hectare (plotted on a log10 scale) at the different sites surveyed. Error bars show ± S.E.M. Bars that share letters are not significantly different from one another. PA = Punta Arena (Sandy control), BS = Bajo del Salado (Rocky control), CPNP = Cabo Pulmo National Park, Reefs = Reef-specific hydroacoustics transects within the CPNP.

There were significantly different acoustic mean densities of fish per hectare between all sites (ANOVA, F_3,68_ = 43.9, P < 0.001). Greatest acoustic fish density was present in the Reef-specific surveys (5388 ± 1282 S.E.M fish/ha) within the park. These were an order of magnitude higher than numbers gained during the standard acoustic survey transects within the CPNP which combined both reef, rocky and sandy habitats (447 ± 141 fish/ha), and higher still in comparison to the control areas (PA = Punta Arena, the sandy control site: 130 ± 40, and BS = Bajo del Salado, the rocky control site: 99 ± 17 fish/ha). Pairwise comparisons between sites showed that CPNP acoustic transects had significantly higher fish density than BS whilst there was no significant difference between BS and PA, or the CPNP and PA (Table 1, Fig. 2). A comparison of fish density inside (CPNP transects excluding the reef-specific surveys) versus outside the reserve (PA and BS) showed fish density was four times higher within the park (T-test, T_51_ = 3.19, P = 0.002).

**Table 1:** Results of Tukey HSD post-hoc tests examining the differences in fish density (log_10_ fish number/ha) between sites. PA = Punta Arena (Sandy control), BS = Bajo del Salado (Rocky control), CPNP = Cabo Pulmo National Park, Reefs = Reef-specific hydroacoustics transects within the CPNP.

Site	PA		Reefs		CPNP	
	T	P	T	P	T	P
Reefs	9.18	<0.001	—	—	—	—
CPNP	2.33	0.102	8.53	<0.001	—	—
BS	0.06	1.00	10.20	<0.001	2.71	<0.001

### Acoustic fish biomass

**Figure 3:**
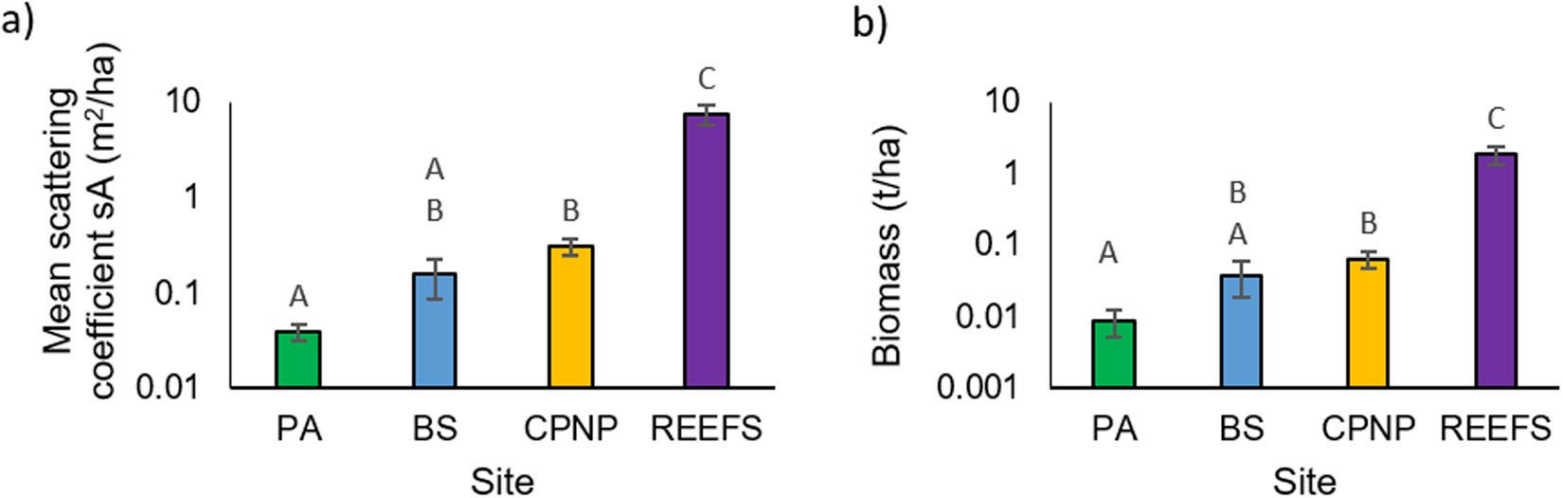
Acoustic Mean values of the area scattering coefficient (sA) (**a**) and fish biomass (**b**) (both plotted on a log10 scale) across the different sites. Error bars show ± S.E.M. Bars that share letters are not significantly different from one another. PA = Punta Arena (Sandy control), BS = Bajo del Salado (Rocky control), CPNP = Cabo Pulmo National Park, Reefs = Reef-specific hydroacoustics transects within the CPNP.

There was a significant correlation between the mean area scattering coefficient (sA) biomass proxy values (the amount of backscattered energy from fish over a given area) and the calculated acoustic fish biomass values for each transect (tonnes/ha) at all sites (Pearson correlation = 0.936, P < 0.001, R^2^ = 0.876). The sA values were also compared to the volume scattering coefficient, linearized s_v_ values (units of m^2^/m^3^) (Pearson correlation = 0.967, P < 0.001, R^2^ = 0.934) demonstrating differences in depth (i.e. volumes esonified) between sites did not have a significant influence on the calculation of sA. The was a significant effect of site on mean sA values (Kruskal-Wallis, H_3_ = 41.79, P < 0.001) (Table 2a, Fig. 3a) as with the log_10_ transformed biomass values (t/ha) (ANOVA, F_3,68_ = 21.75, P < 0.001) (Fig. 3b). Pairwise comparisons between sites showed that reef-specific transects had a significantly higher fish biomass than all other sites and the CPNP and BS had similar values of biomass as PA and BS (see Table 2b below, for details). When both PA and BS were examined together as a general “outside MPA” group, and compared to the CPNP, the biomass values in the CPNP were 273% higher and significantly different for both sA and tonnes per hectare values (Mann Whitney, W = 920.5, P < 0.001; T_51_ = 3.81, P < 0.001, respectively).

**Table 2:** (a) Results of Dunn’s post-hoc tests examining the differences in sA between sites and, (b) Results of Tukey HSD post-hoc tests examining the differences in biomass (log_10_ biomass t/ha) between sites. PA = Punta Arena (Sandy control), BS = Bajo del Salado (Rocky control), CPNP = Cabo Pulmo National Park, Reefs = Reef-specific hydroacoustics transects within the CPNP.

(a) Site	PA		Reefs		CPNP	
	Z	P	Z	P	Z	P
Reefs	5.99	<0.001	—	—	—	—
CPNP	3.46	<0.001	3.5	<0.001	—	—
BS	1.6	0.125	4.88	<0.001	1.94	0.05
**(b) Site**	**PA**		**Reefs**		**CPNP**	
	**T**	**P**	**T**	**P**	**T**	**P**
Reefs	7.46	<0.001	—	—	—	—
CPNP	3.8	0.002	4.92	<0.001	—	—
BS	1.84	0.264	6.23	<0.001	2.03	0.187

### Acoustic fish size

**Figure 4:**
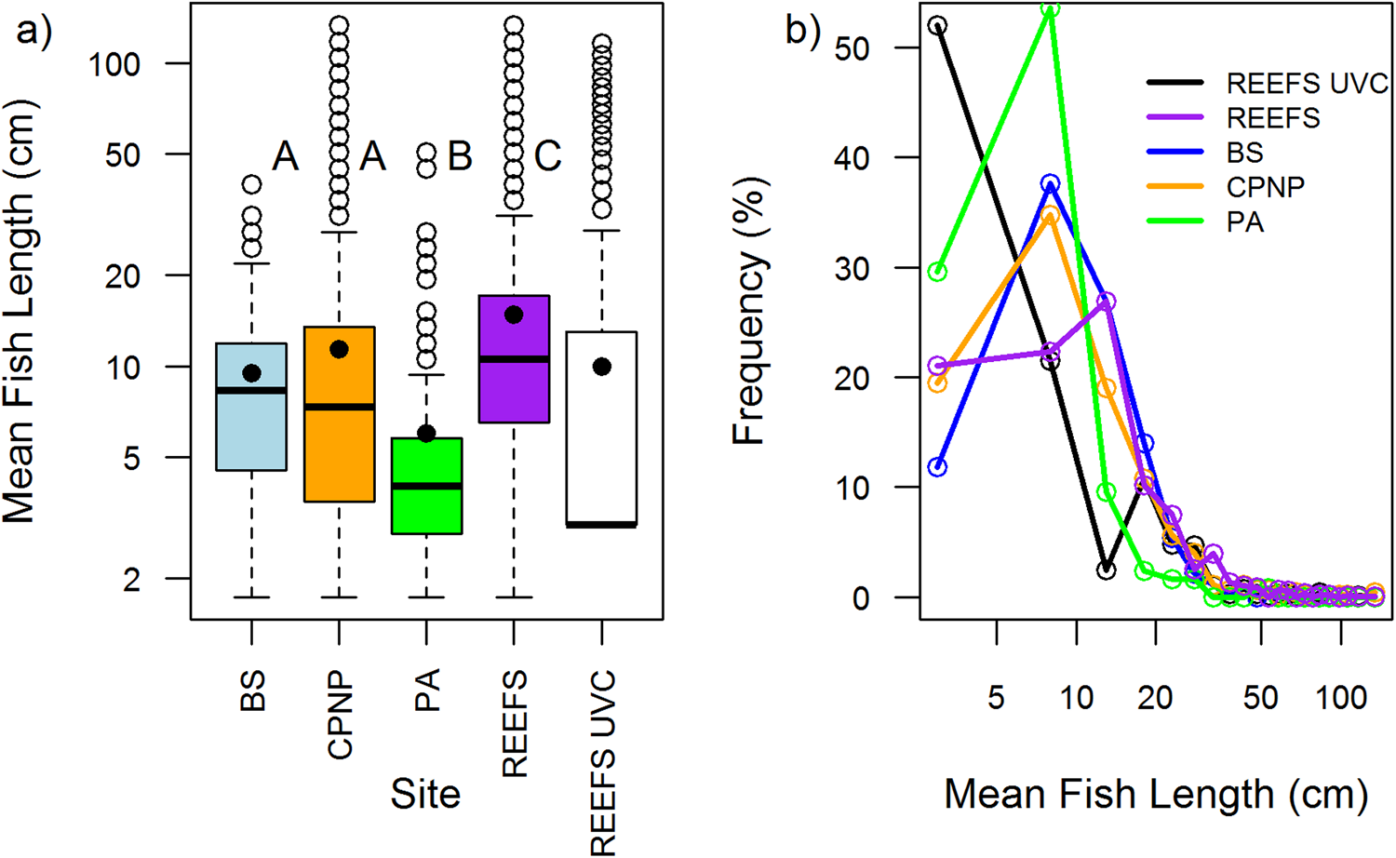
(**a**) Mean fish size (plotted on a log10 scale) at the different survey sites. Sizes in cm gained from converting TS to length through application of the Love (1971) formula. Fish size data from Underwater Visual Census (UVC) surveys of the same reefs in 2015 are shown in the white box and no whisker is present due to the median being in the lowest size class. Bars that share letters are not significantly different from one another (UVC data not included in comparisons). Box plots show mean values (black circle), median values (solid horizontal line), and the lower and upper ends of the box are the 25% and 75% quartiles respectively. The whiskers indicate 1.5 times the inter-quartile range and points beyond this range are shown by empty circles. (**b**) Mean proportions of fish sizes at the different survey sites. Colours in b relate to the sites in a. Data on fish length is plotted on a log10 scale. PA = Punta Arena (Sandy control), BS = Bajo del Salado (Rocky control), CPNP = Cabo Pulmo National Park, Reefs = Reef-specific hydroacoustics transects within the CPNP.

There was a significant difference in the mean size of fish (estimated via acoustics) between sites (Kruskal-Wallis, H_3 = _258.22, P<0.001) (Fig. 4a). Comparisons between sites revealed that the mean size of fish at PA (mean size 6.02 cm ± 0.62) and those over the reef-specific transects (mean size 14.78 cm ± 0.2) were both significantly different from all other sites (Table 3) whilst the mean size of fish inside the CPNP (mean size 11.4 cm ± 0.69) and at BS (mean size 9.5 cm ± 0.71) were not significantly different.

**Table 3:** Results of Dunn’s post-hoc tests on mean fish size as estimated via acoustics between sites. PA = Punta Arena (Sandy control), BS = Bajo del Salado (Rocky control), CPNP = Cabo Pulmo National Park, Reefs = Reef-specific hydroacoustics transects within the CPNP.

Site	PA		Reefs		CPNP	
	Z	P	Z	P	Z	P
Reefs	12.27	<0.001	—	—	—	—
CPNP	5.99	<0.001	10.19	<0.001	—	—
BS	4.56	<0.001	4.66	<0.001	0.16	0.88

### Comparing hydroacoustics and UVC estimates

**Figure 5:**
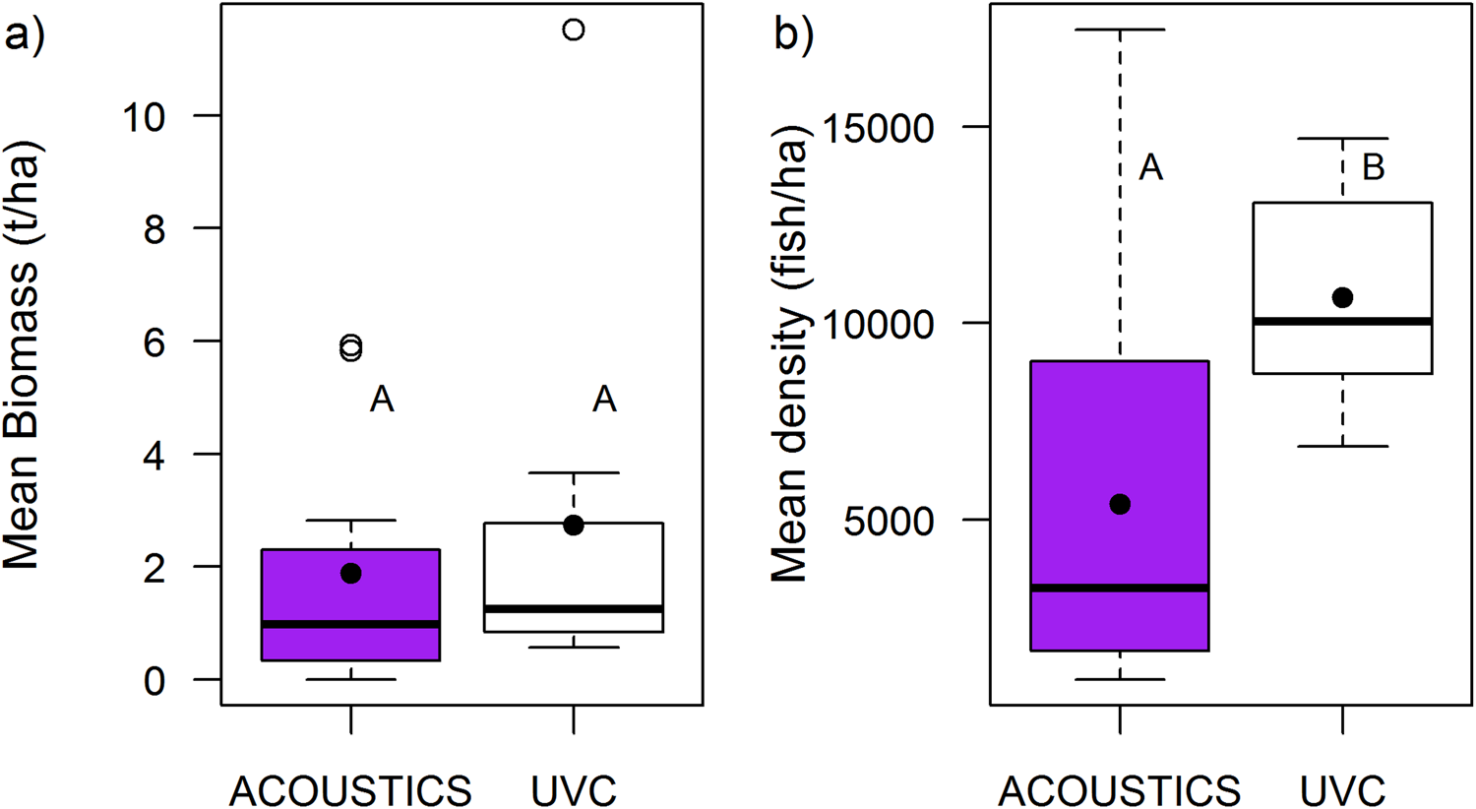
(**a**) Mean fish biomass and (**b**) Mean fish density estimates over the reefs of the Cabo Pulmo National Park (CPNP) from hydroacoustics (purple boxes) and from Underwater Visual Census (UVC) (white boxes) surveys in the same year (2015). Boxes that share letters within plots are not significantly different from one another. Box plots show mean values (black circle), median values (solid horizontal line), and the lower and upper ends of the box are the 25% and 75% quartiles respectively. The whiskers indicate 1.5 times the inter-quartile range and points beyond this range are shown by empty circles.

**Figure 6:**
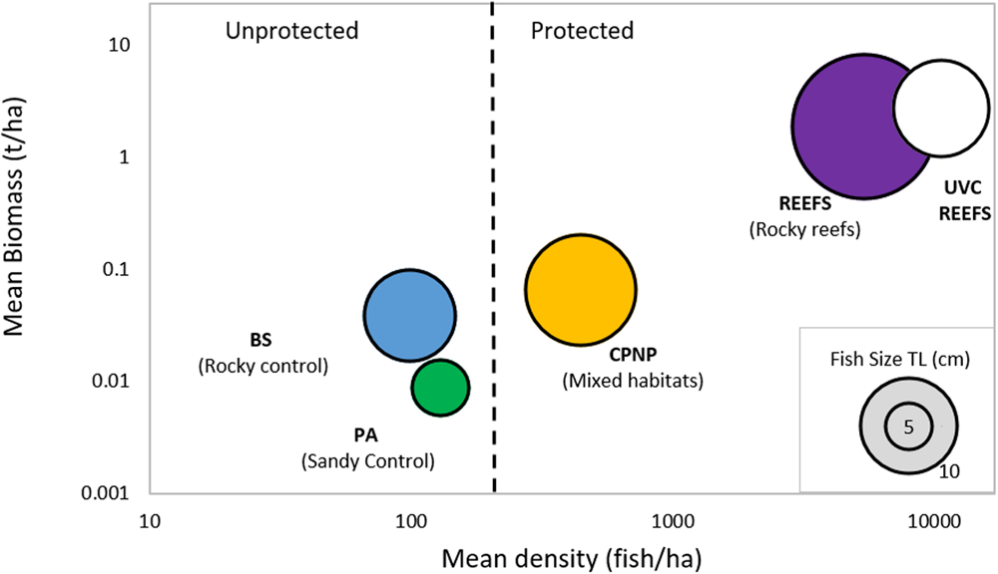
Bubble plot summarising the main findings in this study (x and y axes are log10 transformed). PA = Punta Arena (Sandy control site), BS = Bajo del Salado (Rocky control site), CPNP = Cabo Pulmo National Park, Reefs = Reefs within the CPNP, UVC Reefs = Data for fish collected over the CPNP reefs using Underwater Visual Census (UVC).

There was no significant difference between hydroacoustic median biomass values and UVC median biomass values over the CPNP reefs (Mann-Whitney, W = 46, P = 0.392). Fish density values between these two methods of fish survey were, however, significantly different (Mann-Whitney, W = 23, P = 0.016) (Fig. 5a,b). Fish size over the reef-specific transects was significantly higher from the hydroacoustic survey estimates (median = 8 cm) than the UVC surveys (median = 3 cm) (Mann-Whitney, W = 1.16e^9^, P < 0.001). Further, a significant difference in the shape of the size class distributions was also detected between the two methods (Kolmogorov–Smirnov, KS = 0.309, P = 0.023), with greater numbers of fish in the smaller size classes counted by the UVC surveys. Fish density, biomass and mean fish length data from both the hydroacoustic and UVC surveys are summarised together in Fig. 6.

## Discussion

The literature on both the effects of MPAs as management tools and hydroacoustics for fish surveys is plentiful. To our knowledge, however, this is the first study attempting to investigate the effects of a MPA using hydroacoustics, despite the recognised potential in doing so^39^. Our hydroacoustic surveys showed that there were more, larger fish inside the CPNP than in control areas, outside the park. This is in keeping with most meta-analyses that have reported increased fish density, size and biomass inside MPAs (e.g. Starr *et al*.^40^). The differences in fish community measures (density, biomass and size classes) between the control sites emphasizes the importance of habitat type in determining fish community composition inside versus outside the CPNP^41,42^. It is noteworthy that the greatest fish density, biomass values and mean size were associated with the basaltic rocky reefs within the CPNP. The rocky boulder complex control site (BS) also had more large fish than the sandy bottom control site (PA). Both results highlight the importance of habitat type in addition to protection in determining increased fish density, biomass and size structure, and the utility of using hydroacoustic methods to survey large areas without the associated problems of extractive fishing methods.

Deriving fish density, size and subsequent biomass estimates using *in situ* acoustic Target Strength (TS) is not an exact science^43,44^ as TS is known to vary due to factors such as fish species, aspect, behaviour, condition and maturity^45,46^. However, in a mixed species assemblage such as the CPNP and surrounding waters, it was the only viable option and the same approach has been used previously in similar situations^47–50^. Further imprecision in fish size is likely to have been introduced by using the Love^51^ formula in the conversion of TS to fish size. This may not be suitable for all the fishes within the survey area, but provides a consistent relative scale to describe biological sizes of the fish community across all of the sample sites^47,52,53^. The values derived from this should therefore be considered as an approximation as species specificity was not possible due to the highly diverse fish community and lack of species-specific conversion formulae^44^. Whilst the scattering coefficient sA is commonly used as a proxy for biomass, t/ha values were also calculated to allow comparison with the diver based UVC estimates. The t/ha units are also more convenient and interpretable than the original units of m^2^/ha^47^. This type of conversion has previously been undertaken for mixed species communities by taking mean ‘a’ and ‘b’ values in the W-L formula for the species present (e.g. Wanzenböck *et al*.^49^; Boswell *et al*.^47^). We took this idea further by applying general values for ‘a’ and ‘b’ from Froese^54^. Whilst the imprecision from using this general length-weight formula is acknowledged, a strong positive correlation between our t/ha values and the m^2^/ha sA values indicates the general viability in our calculated t/ha values for fish biomass.

To fully determine the effects of MPA placement on local fish populations, a Before, After, Control, Impact (BACI) design is necessary^26,55,56^, with the establishment of the park as the ‘Impact’. Underwood^57^ states that more than a single control is necessary to reduce the likelihood of coincidental change; ideally at least 2 sites with each combination of habitat and protection would be required to more fully determine protection effects^58^. The choice of such independent control sites is, however, difficult in a heterogeneous environment and poses considerable logistic and financial constraints^6,59^, but is recommended in future studies adopting a survey approach similar to our own. When evaluating the effects of an MPA, care must be taken so that protected area effects are not exaggerated or masked by other effects such as habitat^7,8^, as MPAs are often placed in particularly rich and/or abundant habitats^60^. Whilst it was not possible to survey the CPNP using our hydroacoustic approach prior to its implementation, we were able to provide two control sites that were open to fishing that contained the predominant habitat types found within the CPNP. The main combinations of habitat as well as exposure to fishing (protected *vs* not protected) were therefore accounted for in our survey design, however investigations into habitat effects were investigated further by examining the fish associated with the reefs present within the CPNP separately from the park as a whole. For an ideally balanced design we would have had transects from outside the park over similar reefs structures, however, this was not possible as none are present in the neighbouring areas.

The overall higher fish density and biomass across the CPNP is potentially a result of a “spillover” effect^61–64^ occurring from significantly higher densities of fish associated with the reefs inside the park. In turn, it is possible that a spillover effect from the CPNP may have also increased fish density, biomass and size at the control sites. Although the whole park is effectively managed as a no-take area by the local community^35^, there is unfortunately no data available on the levels of fishing at the control sites, although active fishing activity was observed at both locations during our surveys. The high density of small fish at PA may be due to high levels of size-selective fishing practices occurring here rather than, or in combination with, any habitat effects. This would have the effect of leaving a greater proportion of smaller fish in an area and also increasing the number of prey (a “prey release”) following removal of the larger predators^34,65–67^. This, however, requires further investigation using fisheries data from these areas, as well as more detailed hydroacoustic surveys with a temporal aspect to capture changes in fishing behaviours and fish densities.

The relationship between marine fish species and their habitats is a key component in understanding their distributions^68^. Further, habitat complexity has long been known to have a positive effect on fish abundance^27,29,69,70^ and biomass^28^. Our results agree with such findings that the most complex habitat, in this case the basalt reefs inside the CPNP, yield the highest fish density, biomass and mean size. Increased habitat complexity has been shown to have a strong positive effect on adult fish density and a weaker effect on recruit abundance^71^. Using hydroacoustics, Boswell *et al*.^47^ found significantly smaller fish over sandy habitats in comparison to more rugose habitats. The effect of habitat complexity may therefore be more pronounced with certain size classes of fish^72^. Excluding the reef specific transects, the highest fish density, biomass and size values were found within the CPNP area which is composed of a mix of the predominant habitats found at the control sites: heterogeneous sand/boulder/reef habitats. Between the control sites, greater numbers of fish (but not biomass) were present at PA compared to BS, a surprising result as BS contains a complex rocky habitat more likely to favour higher fish biomass similar to the rocky areas within the park. However, this demonstrates how small fish do not contribute greatly to biomass levels at PA, despite relatively high densities^49^. The higher fish density, biomass and mean fish size present over the reef specific transects could also be due to the generally shallower depths of these in comparison to the other sites. In other locations the overall and relative abundances of different trophic groups of fishes has been described at different depths^73,74^. The detailed analysis of fish distribution with depth was beyond the scope of the study, due to the large variation in depth along transect, and this is another reason why it was appropriate to separate these reef-specific sites out from the analysis of the CPNP vs control sites. In the examination of the CPNP vs control sites the mean water depths investigated along transects were, however, similar.

Our density values differed significantly from those of the UVC surveys from the same reefs in the same year, although our biomass values did not. Examining the size class distributions resultant from the two methods, this can be explained by the UVCs recording more fish in the smaller size classes than our hydroacoustic methods. Acoustics should not be biased in detecting these smaller fish when they are sufficiently separated from the reef matrix. It is, however likely that many of these small individuals are more cryptic and substrate-affiliated in nature than larger fishes^75^. Our density and biomass values, are therefore likely to be conservative as smaller fish with closer associations with the seabed (within the “acoustic dead zone”) will likely not have been counted by our hydroacoustic methods^50,76^. Further, it is likely that this effect will have been more pronounced in areas of more complex habitat such as the reefs and boulders and if areas with overhangs and caves are present, then fish densities would certainly be underestimated.

Differences in fish density estimates could also be caused by potential differences in the precise locations of diver surveys on the reefs compared to the hydroacoustic transects. Differences in fish avoidance behaviour between the acoustic survey vessel and survey divers could also explain some differences in density estimates^77,78^). Finally, it is possible that differences in fish densities between the UVC and acoustic surveys may be caused by temporal variability. Both UVC and hydroacoustics were, however, conducted during daylight hours (avoiding crepuscular periods). It should however be noted that both the hydroacoustic and UVC datasets represent snapshots in time and further interseasonal and interannual surveys would be beneficial. Little seasonal variation in the fish assemblages over the Cabo Pulmo reefs has, however, been noted^79^.

Overall our acoustic survey campaign took one researcher and one boat operator a total of 8 days to survey the whole park twice as well as the two control sites and the final reef-specific surveys inside the CPNP. Hydroacoustics have the capacity to cover a greater area in a similar amount of time compared to UVC surveys. Additionally, acoustics are not constrained by issues such as poor water clarity, strong currents or diver depth limits which can make some areas impossible for SCUBA surveys. Both hydroacoustic and UVC methods, however, can be hampered by adverse sea states^80^. The start-up costs for the hydroacoustic equipment may be an impediment to their adoption for MPA evaluations, as we estimate they are approximately double that of an equivalent UVC SCUBA team (including training, certification and equipment). The UVC surveys in the CPNP took 4 divers, 6 days to survey 12 reefs within the park which corresponds approximately to 0.1% of the total park area. UVC, however, can provide high-resolution, species-specific information from which subtler ecosystem shifts than changes in overall measures (e.g. density, biomass, size) can potentially be detected^36,81^. UVC surveys can also give additional information on MPA performance such as habitat health and invertebrate surveys which cannot be assessed through the hydroacoustic method we present here. Further, UVC will provide more detail on demersal species whilst hydroacoustics gives more information throughout the water column. We therefore conclude that there is considerable merit in nesting UVC surveys within a hydroacoustic survey campaign, to provide higher resolution species-specific information in conjunction with the broader scale estimates of fish density, biomass and size (see also Murphy and Jenkins^82^).

Our hydroacoustic surveys revealed important information on the nature of fish distributions inside, outside and amongst the differing habitats of the CPNP. This study highlights the importance of both protection and habitat in producing high fish density, biomass and mean sizes, emphasising the need to account for differences in habitat when designing coastal MPAs. Hydroacoustic surveys represent a valuable, non-invasive tool for the assessment of MPA fish populations, something that until now has been underutilised in MPA formation and management.

## Methods

### Field surveys

Hydroacoustic field surveys in and around the CPNP were undertaken during March 2015 in collaboration with a local SCUBA diving company (Cabo Pulmo Divers). All survey protocols were approved by Comisión Nacional de Áreas Naturales Protegidas (CONANP).

To undertake hydroacoustic fish surveys, there needs to be adequate coverage over the survey areas to gain a reliable picture of the local fish distributions. Degree of coverage (Λ) is defined as:

1 $${\rm{\Lambda }}=D/\sqrt{A}$$

where: *D* is the cruise track length; and, *A* is the size of the survey area^83^, and for adequate coverage the ratio needs to be ≥6. This was achieved in all the different survey sites. Control sites outside the park and therefore open to fishing were: 1) Punta Arena (PA), a mainly sandy bottom site located 5 km to the north of the CPNP; and 2) Bajo del Salado (BS), a boulder-reef complex 5 km to the south. High resolution reef-specific surveys were also undertaken inside the CPNP by running hydroacoustic transects along each discrete reef area (Fig. 1, Table 4.). These reef areas were located using the local knowledge of the skipper and previous SCUBA monitoring of the sites. On all reef-specific transects, bottom type was confirmed using a towed camera system to ensure that the reefs were being correctly targeted. The towed camera system was also used to identify groups of fish species within transects where possible. Overall, the whole hydroacoustic survey campaign took one researcher and one boat operator a total of 8 days to survey the whole 7,111 ha park (with double the necessary coverage), the two control sites and the final reef-specific survey within the CPNP.

**Table 4:** Descriptions of the survey sites summarising substrate types, mean depths and protection afforded.

Site name	Abbreviation	Habitat/Substrate type	Mean Depth (m) of area investigated (±S.D)	Protect-ion
Cabo Pulmo Nat. Park	CPNP	Sand, boulders & rocky reefs	72.6 ± 23.5	✓
Punta Arena	PA	Mainly sand	86.9 ± 14.9	X
Bajo Del Salado	BS	Boulder-reef complex	59.8 ± 24.9	X
Reef specific	—	Rocky basaltic reefs	12.3 ± 4.4	✓

### Acoustic Equipment

A Biosonics® DTX Split beam echosounder with a 200 kHz transducer was used for the surveys, pole mounted over the side of the survey vessel with the transducer face 1 m below the water surface. Acoustic data were georeferenced with an integrated Garmin 17Xhvs GPS, and a laptop computer loaded with Biosonics acquisition software (Visual Acquisition, 2010). The circular transducer used with this system has a beam-opening angle of 6.8° (3 dB beam width). Pulse duration was 0.4 ms with a specified max ping rate of 10 per second. Survey speed throughout the surveys was kept under 6 knots. Calibration used a standard Biosonics 36 mm Tungsten Carbide 200 kHz calibration sphere before the surveys, following the standard methods of Foote *et al*.^84^.

### Acoustic Data processing

Data were collected as DT4 files and then converted and post-processed with the Sonar5-Pro software package^85^. Analysis followed the Software Guided Analysis (SGA) routine based upon the standard operating procedure of Parker-Stetter *et al*.^86^. Density estimates were calculated by echo integration (EI), which divides the average reflection from all fish over a specified volume (the volume backscattering coefficient, s_v_) by the average backscattering cross section (σ_bs_) which is derived from the mean echo intensity (Target Strength (TS)) from individual fish^87,88^. These TS values were obtained *in situ* and based on Single Echo Detections (SED). SEDs had a minimum echo length of 0.8 dB, a maximum of 1.2 dB and a maximum angle standard deviation of 0.8 degrees. Multi-peak suppression was set to ‘medium’ in the Sonar5 software which requires a dip of 1.5 dB between peaks if the echo is to be rejected. Thresholds of −60 dB for TS values, and −66 dB for s_v_ values were applied to the data to initially discern fish from other particulate targets such as plankton^89^. To ensure that no echoes from the seabed were classified as fish^76^, a bottom layer of 1 m was applied and any returns from this layer were removed unless they could be clearly identified as fish with definite separation visible between the return and the seabed. Similarly, a surface layer of 1 m was applied to remove surface noise from wind and wave action. On rare occasions this noise had to be removed to 5 m due to abnormally poor surface conditions which was undertaken manually. The data were processed up to a depth limit of 100 m as beyond this the acoustic signal to noise ratio (SNR) became unacceptably low^90^. To compensate for changes in echo intensity due to increasing range (R), a Time Varied Gain (TVG) of 40 log(R) for TS values and 20 log(R) for s_v_ values was used as recommended in Sonar5^85^. Whole transects were taken as Elementary Sampling Distance Units (EDSU’s) to maximize the number of EDSUs with sufficient SED as the source of *in situ* TS for the calculations^85^. To minimize potential spatial autocorrelation, we calculated s_v_ for the entire esonified water volume^91^. Mean TSs were checked for multiple echo bias following Sawada *et al*.^92^ and each EDSU had a fish per esonified volume (Nv) less than 0.1^43^.

### Acoustic Fish Size

TS is an indicator of fish size but is also influenced by species, due to differences in ratios of body size to bladder size^44^, and swimming behaviour (e.g. tilt angle) of the species or individual^93^. To translate TS into more intuitive length measurements (cm) than the decibel^47^ it is converted using empirical TS-length relationships, which often exist for specific groups or species^39^. There was a wide diversity of fish species in the area making the use of species- specific TS–length formulas problematic^94^. Further, for most of the species identified using a towed underwater camera, empirical TS-length formulae are yet to be established. It was therefore necessary to apply a multi-species equation derived from Love^51,95^ to convert TS to length with the following conversion:

2 $${\rm{TS}}=(19.1\,{\mathrm{log}}_{10}{\rm{L}})-(0.9\,{\mathrm{log}}_{10}{\rm{f}})-62.0$$

where TS = target strength detected (dB), L = length (Total Length) of the target (cm), and f = the frequency used. With a transducer frequency of 200 kHz (as used in these surveys) this equation then becomes:

3 $${\rm{TS}}=19.1\,{\mathrm{log}}_{10}({\rm{L}})-64.07$$

Reformulation of Equation 3 to gain unknown lengths from known TS therefore becomes:

4 $${\rm{L}}={10}^{((\text{TS}+64.07)/19.1)}$$

### Acoustic Biomass

Total fish biomass was also examined as it provides a better measure of productivity than fish density values^96^. Commonly in hydroacoustic surveys, the scattering coefficient (sA) presented in terms of an area (m^2^/ha) is used as a proxy for biomass^44,48^. It quantifies the amount of a unit area occupied by fish, considering the water depth^94^. We compared these sA units between sites and sA values was also compared to linearized s_v_ values (units of m^2^/m^3^) to ensure differences in depth between sites were not having a significant influence on this parameter. To compare hydroacoustic biomass values with those estimated from UVC surveys, it was also necessary to calculate biomass values with units of t/ha following Yurista *et al*.^17^. To do this, 5 different steps were undertaken: 1) TS distributions (based on SED from −60 dB to −20 dB in 1 dB bins) for each transect were converted to fish length by using the aforementioned multispecies equation from Love^51^. 2) The midpoint of these length bins was then converted to weight by using a generalist W-L equation gained from a meta-analysis by Froese^54^:

5 $${\rm{W}}={\rm{a}}\times {{\rm{L}}}^{{\rm{b}}}$$

(constants a=0.0137 and b=3 and L is Total Length). 3) The proportions of the different weights present in each sample were then multiplied by the total density values (# fish/ha) of each transect, giving the number of fish per 5 cm weight class. 4) These values were summed per weight class to give a biomass value in t/ha. 5) The average biomass value per site was then calculated as the arithmetic mean of all transects within each site.

### UVC Surveys

We took advantage of the monitoring program that has been undertaken over the reefs within the CPNP during the months of August and September since 2000^36,97–99^. Under this program, UVC surveys using SCUBA are conducted using the standard methods for visual belt transects^100^. A total of six divers count and identify all fishes observed to species level at each reef site. UVC data from 8 reefs (those in the same locations as the hydroacoustic reef surveys) were used for comparison with the hydroacoustic data we collected during the field campaign. At each site, a two-person dive team survey 50 m transects counting and estimating the sizes of all fish and invertebrates, within a 5 m wide belt along each transect during two passes. This results in between 4 and 8 replicates per each 250 m^2^ total area. Mobile species versus territorial species are surveyed in separate passes, to ensure that the same individuals are only counted once. A table of fish species recorded from the UVC surveys over the reefs, with densities, trophic group, mean sizes and biomasses is provided in Appendix S1.

### Statistical analyses

A large school of jacks (*Caranx Sexfasciatus*) (9 m high by 25 m long) was encountered during the reef-specific acoustic transects, this stochastic event created a significant outlier in the data (increasing mean acoustic ‘reef-specific’ density values by 20%). There are also many difficulties in calculating density estimates for dense fish schools due to sound attenuation^44^. We therefore excluded this from all further analyses meaning our estimates of fish density and biomass for the reef-specific surveys were conservative. Mean values (±S.E.M) of fish density, biomass and size were calculated from the two surveys inside the park as there was no significant difference between these surveys for any of the parameters. This also highlights the repeatability of the method we employed. Biomass values derived from TS values were compared with sA values via ordinary least squares (OLS) regression. To compare fish density, biomass, sA and length data between the different sites, Welches one-way ANOVAs were used. Following each ANOVA, Tukey’s post-hoc multiple comparisons were performed to determine where any significant differences between sites occurred. If assumptions of normality or equal variance were not met, then the data were first log_10_ transformed. If data transformation did not address the violations of normality, a Kruskal-Wallis test was used on the non-transformed data followed by Dunn’s post-hoc tests. Two-sample T-tests (or where necessary the non-parametric equivalent Mann-Whitney) were then also used to test differences in mean fish density, biomass, sA and fish size between the CPNP transects and all control transects. Mean fish size and biomass data from SCUBA UVC surveys undertaken in 2015 were compared with our reef-specific fish data using Mann-Whitney tests and fish size class frequency distributions by a 2 sample Kolmogorov–Smirnov test following reassignment of acoustic data to the fish size classes given by UVC surveys.

## Electronic supplementary material

Appendix S1
