# Supplementary material for: Hydroacoustics as a tool to examine the effects of Marine Protected Areas and habitat type on marine fish communities
Source: Sci Rep. 2018 Jan 15;8:47. doi: 10.1038/s41598-017-18353-3 (PMC5768732; doi:10.1038/s41598-017-18353-3)
Supplement: Supplementary file 1 — Appendix S1 [file 41598_2017_18353_MOESM1_ESM.doc]

**Hydroacoustics as a tool to examine the effects of Marine Protected Areas and habitat type on marine fish communities.**

J.P. Egerton^1^, A.F. Johnson^2^*, J. Turner^1^, L. Le Vay^1^, I. Mascareñas-Osorio^3^, O. Aburto-Oropeza^2^

^1^School of Ocean Sciences, Bangor University, Menai Bridge, Wales, UK. [j.egerton@bangor.ac.uk](mailto:osp23e@bangor.ac.uk)

^2^Marine Biology Research Division, The Scripps Institute of Oceanography, California, US.

*corresponding author: [afjohnson@ucsd.edu](mailto:afjohnson@ucsd.edu)

^3^Centro para la Biodiversidad y Conservación, La Paz, México.

**Appendix S1.**

Table of fish species recorded from the 2015 UVC surveys over the reefs in the Cabo Pulmo National Park, with total abundances, trophic group, mean sizes and biomass.

| Species | Abundance  (# fish) | Mean size (cm) | Size  (±S.E.M) | Biomass (tonne/ha) |
| --- | --- | --- | --- | --- |
| Carnivores | | | | |
| *Anisotremus taeniatus* | 65 | 29.92 | 0.8 | 0.174 |
| *Arothron meleagris* | 77 | 20.71 | 0.53 | 0.023 |
| *Balistes polylepis* | 11 | 12.73 | 2.17 | 0.003 |
| *Bodianus diplotaenia* | 236 | 13.62 | 0.56 | 0.028 |
| *Canthigaster punctatissima* | 338 | 5 |  | 0.001 |
| *Chaetodon humeralis* | 10 | 20 |  | 0.027 |
| *Chanos chanos* | 74 | 80.27 | 1.15 | 8.833 |
| *Cirrhitichthys oxycephalus* | 1,039 | 5.13 | 0.03 | 0.002 |
| *Dasyatis dipterura* | 1 | 60 |  | 0.002 |
| *Decapterus muroadsi* | 100 | 10 |  | 0.011 |
| *Diodon holocanthus* | 42 | 21.07 | 0.81 | 0.018 |
| *Diodon hystrix* | 5 | 48 | 1.22 | 0.072 |
| *Epinephelus labriformis* | 70 | 24.14 | 0.64 | 0.031 |
| *Forcipiger flavissimus* | 1 | 15 |  | 0.001 |
| *Gymnothorax castaneus* | 11 | 103.82 | 9.74 | 0.061 |
| *Halichoeres chierchiae* | 6 | 14.17 | 1.54 | 0.001 |
| *Halichoeres dispilus* | 288 | 10.5 | 0.32 | 0.009 |
| *Halichoeres melanotis* | 4 | 17.5 | 2.5 | 0.001 |
| *Halichoeres nicholsi* | 1 | 25 |  | 0.003 |
| *Halichoeres notospilus* | 3 | 25 |  | 0.003 |
| *Johnrandallia nigrirostris* | 66 | 13.11 | 0.5 | 0.007 |
| *Lutjanus viridis* | 77 | 22.6 | 0.33 | 0.029 |
| *Mulloidichthys dentatus* | 111 | 22.66 | 0.48 | 0.044 |
| *Muraena lentiginosa* | 1 | 35 |  | 0.001 |
| *Novaculichthys taeniourus* | 1 | 25 |  | 0.002 |
| *Ostracion meleagris meleagris* | 9 | 10.56 | 1.3 | 0.001 |
| *Plagiotremus azaleus* | 133 | 6.88 | 0.21 | 0.0003 |
| *Pomacanthus zonipectus* | 1 | 30 |  | 0.008 |
| *Pseudobalistes naufragium* | 4 | 52.5 | 5.2 | 0.058 |
| *Rypticus bicolor* | 2 | 7.5 | 2.5 | 0.0001 |
| *Serranus psittacinus* | 36 | 12.22 | 0.81 | >0.0001 |
| *Sufflamen verres* | 124 | 16.33 | 0.78 | 0.025 |
| *Thalassoma grammaticum* | 231 | 7.45 | 0.35 | 0.005 |
| *Thalassoma lucasanum* | 2,520 | 6.81 | 0.17 | 0.144 |
| *Zanclus cornutus* | 52 | 14.52 | 0.63 | 0.015 |
| Herbivores | | | | |
| *Acanthurus nigricans* | 16 | 18.13 | 1.43 | 0.007 |
| *Acanthurus xanthopterus* | 57 | 28.77 | 1.06 | 0.072 |
| *Holacanthus clarionensis* | 1 | 10 |  | 0.0003 |
| *Holacanthus passer* | 135 | 17.11 | 0.63 | 0.038 |
| *Kyphosus analogus* | 8 | 37.5 | 0.94 | 0.013 |
| *Kyphosus elegans* | 1 | 30 |  | 0.005 |
| *Microspathodon dorsalis* | 6 | 12.5 | 3.59 | 0.003 |
| *Nicholsina denticulata* | 1 | 20 |  | 0.001 |
| *Ophioblennius steindachneri* | 67 | 13.36 | 0.59 | 0.004 |
| *Prionurus punctatus* | 415 | 25.1 | 0.26 | 0.27 |
| *Scarus compressus* | 4 | 38.75 | 9.44 | 0.031 |
| *Scarus ghobban* | 23 | 45 | 3.16 | 0.058 |
| *Scarus perrico* | 3 | 31.67 | 6.01 | 0.011 |
| *Scarus rubroviolaceus* | 26 | 43.08 | 3.27 | 0.066 |
| *Stegastes flavilatus* | 366 | 7.1 | 0.13 | 0.004 |
| *Stegastes rectifraenum* | 630 | 8.02 | 0.11 | 0.01 |
| Piscivores | | | | |
| *Aulostomus chinensis* | 1 | 10 |  | >0.0001 |
| *Carangoides orthogrammus* | 8 | 35.63 | 1.48 | 0.032 |
| *Caranx caballus* | 10 | 31.5 | 1.5 | 0.063 |
| *Caranx sexfasciatus* | 50 | 45 |  | 1.217 |
| *Cephalopholis panamensis* | 44 | 19.66 | 1.39 | 0.013 |
| *Cirrhithus rivulatus* | 4 | 22.5 | 2.5 | 0.005 |
| *Fistularia commersonii* | 18 | 96.11 | 9.35 | 0.045 |
| *Gnathanodon speciosus* | 2 | 35 |  | 0.02 |
| *Hoplopagrus guentherii* | 4 | 43.75 | 5.15 | 0.043 |
| *Lutjanus argentiventris* | 20 | 50.5 | 2.11 | 0.5 |
| *Lutjanus novemfasciatus* | 4 | 65 | 5 | 0.168 |
| *Mycteroperca rosacea* | 121 | 48.55 | 1.15 | 0.357 |
| Zooplanktivores | | | | |
| *Abudefduf troschelii* | 16 | 18.44 | 1.09 | 0.02 |
| *Chromis atrilobata* | 2,039 | 6.32 | 0.05 | 0.009 |
| *Myripristis leiognathus* | 95 | 9.26 | 0.64 | 0.021 |
| *Paranthias colonus* | 917 | 20.75 | 0.23 | 0.108 |
